# Lactate‐Driven Restriction of Mitochondrial Permeability Transition Promotes Resistance to Chemo‐Immunotherapy by Suppressing Tumor PANoptosis

**DOI:** 10.1002/advs.76321

**Published:** 2026-06-30

**Authors:** Sen Zhong, Wenlong Chen, Fanglong Liu, Shengyi Zhou, Bolin Yu, Yuying Wang, Xiqian Zhou, Xuehui Wang, Diya Liu, Zhuoyu Zhang, Xinran Wang, Mounia Lalouly, Yiwen Li, Zhefei Du, Tao Yan, Zhihui Xiao, Zhou Zhou, Huanhuan Zhu, Fengyuan Qian, Bowen Zheng, Lin Fang

**Affiliations:** ^1^ School of Medicine Shanghai Tenth People's Hospital Department of Breast and Thyroid Surgery Tongji University Shanghai P. R. China; ^2^ Department of Thyroid and Breast Surgery Shanghai Tenth People's Hospital Clinical Medical College of Nanjing Medical University Shanghai P. R. China; ^3^ Department of Breast Surgery Xinhua Hospital Affiliated to Shanghai Jiao Tong University School of Medicine Shanghai P. R. China; ^4^ Department of Breast Surgery The First Affiliated Hospital of Anhui University of Science and Technology Huainan P. R. China; ^5^ Division of Spine Department of Orthopedics Tongji Hospital affiliated to Tongji University School of Medicine Shanghai P. R. China; ^6^ Ministry of Education Key Laboratory of Spine and Spinal Cord Injury Repair and Regeneration (Tongji University) Shanghai P. R. China; ^7^ Translational Research Institute of Brain and Brain‐Like Intelligence Shanghai Fourth People's Hospital, and Cancer Center School of Medicine Tongji University Shanghai P. R. China; ^8^ Department of Breast Surgery Zhejiang Cancer Hospital Hangzhou Institute of Medicine (HIM) Chinese Academy of Sciences Hangzhou Zhejiang P. R. China; ^9^ Wenzhou Medical University Wenzhou Zhejiang P. R. China

**Keywords:** chemo‐immunotherapy resistance, lactate metabolism, lactylation, mitochondrial permeability transition, PANoptosis

## Abstract

Intrinsic resistance limits chemo‐immunotherapy efficacy in triple‐negative breast cancer (TNBC). While metabolic reprogramming is linked to immune evasion, the precise mechanistic orchestration remains unclear. Here, utilizing single‐cell transcriptomics and quantitative lactylome profiling, we show that elevated tumor lactate drives resistance by broadly suppressing PANoptosis. Mechanistically, under chemotherapeutic stress, the Lysine acetyltransferase 8 (KAT8) catalyzes the specific lactylation of the inner mitochondrial membrane ADP/ATP translocator 2 (ANT2) at K92. Lactylated ANT2 recruits the phosphoglycerate mutase 5 (PGAM5) to dephosphorylate Cyclophilin D (CypD). This cascade restricts mitochondrial permeability transition pore (mPTP) opening, preserving mitochondrial homeostasis and averting immunogenic cell death. Crucially, a cell‐penetrating competitive peptide targeting the KAT8‐ANT2 interface effectively uncouples this metabolic lock, re‐sensitizing TNBC tumors to cytotoxic stress and restoring chemo‐immunotherapy efficacy in vivo. Our findings unveil a profound mechanistic link between the Warburg effect and mitochondrial homeostasis, establishing KAT8‐mediated ANT2 lactylation as a targetable vulnerability to improve chemo‐immunotherapy efficacy.

## Graphical abstract

Tumor‐derived lactate establishes a metabolic lock that drives chemo‐immunotherapy resistance by suppressing PANoptosis. Mechanistically, KAT8 lactylates the mitochondrial translocator ANT2, which recruits PGAM5 to dephosphorylate CypD. This cascade restricts mitochondrial permeability transition pore opening, preventing pro‐inflammatory mtDNA leakage. A targeted peptide uncouples this axis, exposing a novel vulnerability to overcome immune evasion.

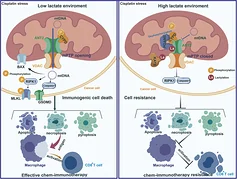

## 1 Background

Immune checkpoint blockade (ICB) combined with chemotherapy has established a new standard of care for advanced triple‐negative breast cancer (TNBC) [1, 2, 3, 4]. The therapeutic efficacy of this regimen fundamentally relies on the ability of cytotoxic agents, such as platinum drugs, to trigger immunogenic cell death (ICD), such as PANoptosis, which facilitates the release of damage‐associated molecular patterns (DAMPs) that elicit ICD‐like inflammatory consequences, inciting localized inflammation and priming the tumor immune microenvironment (TME), thereby sensitizing tumors to ICB [5, 6, 7]. At the core of this cytotoxic response is the mitochondrial permeability transition (mPT). Chemotherapy‐induced oxidative stress provokes the opening of the cyclophilin D (CypD)‐dependent mPT pore (mPTP), precipitating mitochondrial depolarization, respiratory collapse, and the catastrophic cytosolic leakage of pro‐inflammatory mitochondrial contents [8, 9, 10, 11]. Consequently, preserving mitochondrial integrity under cytotoxic stress is a prerequisite for tumor cells to evade PANoptosis and subvert immunotherapy.

To withstand these extreme environmental and therapeutic pressures, tumor cells universally engage in metabolic reprogramming. A hallmark of this adaptation is the massive accumulation of intracellular lactate. Historically viewed as a metabolic endpoint, lactate is now recognized as a potent signaling hub and epigenetic modulator [12, 13, 14, 15, 16, 17]. Within the TME, extracellular lactate drives a highly immunosuppressive niche by inducing tissue‐resident NK cell apoptosis [18], polarizing macrophages via the GPR132–AMPK axis [19], driving dendritic cell tolerogenesis [20], and upregulating PD‐1 on CD8^+^ T cells [21]. However, while these receptor‐mediated and paracrine functions are well defined, it remains entirely unknown whether intracellular lactate mechanically fortifies tumor mitochondria against therapy‐induced mPT to intrinsically restrict ICD.

Here, we identify a direct structural mechanism by which tumor‐derived lactate dictates resistance to chemo‐immunotherapy in TNBC. We show that in patients refractory to combined therapy, elevated glycolytic flux and subsequent lactate accumulation intrinsically restrict mPTP opening under chemotherapeutic stress. Mechanistically, lactate serves as a direct substrate for the lysine acetyltransferase KAT8, which specifically lactylates the inner mitochondrial membrane translocator ANT2 at residue K92. This post‐translational modification transforms ANT2 into a scaffold that recruits the phosphatase PGAM5. PGAM5 subsequently dephosphorylates CypD at the mitochondrial matrix face, raising the gating threshold of the mPTP against oxidative stress. This lactate‐driven “metabolic lock” effectively prevents mitochondrial collapse and immunogenic DAMP leakage, thereby shielding tumor cells from PANoptosis and abrogating the efficacy of chemo‐immunotherapy.

## 2 Methods

### 2.1 Human Tissue Samples

Human breast tumor tissue specimens were obtained from the Department of Thyroid and Breast Surgery, Shanghai Tenth People's Hospital, affiliated with Tongji University (Shanghai, China). Written informed consent was obtained from all participants prior to tissue collection. The study protocol was approved by the Institutional Ethics Committee of Shanghai Tenth People's Hospital, and all procedures were conducted in strict accordance with the ethical principles formulated in the Declaration of Helsinki.

### 2.2 Cell Lines, Cell Culture, and Treatments

Human embryonic kidney (HEK) 293T cells and human triple‐negative breast cancer (TNBC) cell lines (MDA‐MB‐231 and BT‐549) were purchased from the Chinese Academy of Sciences (Shanghai, China) in 2022. MDA‐MB‐231 and HEK293T cells were cultured in high‐glucose Dulbecco's Modified Eagle Medium (DMEM; Gibco, USA). BT‐549 cells were maintained in RPMI‐1640 medium (Gibco, USA). All culture media were supplemented with 10% fetal bovine serum (FBS; Gibco) and 1% penicillin–streptomycin solution (Enpromise, China). Cells were maintained in a humidified incubator at 37°C with 5% CO_2_. All cell lines used in this study were routinely tested for mycoplasma contamination and authenticated to ensure optimal growth conditions. Plasmids were transfected into cells using the Lipo8000 transfection reagent in strict accordance with the manufacturer's protocol. All plasmids were purchased from MiaoLingBio (Wuhan, China). The specific target sequences for the knockdown and knockout plasmids are detailed in Table S5.

### 2.3 Western Blot

Extracted whole‐cell proteins were denatured in 1× SDS loading buffer, resolved by SDS‐PAGE (polyacrylamide gel electrophoresis), and subsequently transferred onto nitrocellulose membranes. The membranes were blocked with 5% non‐fat milk or bovine serum albumin (BSA) and then incubated with the indicated primary antibodies overnight at 4°C. Following incubation with the appropriate secondary antibodies for 1 h at room temperature, the protein bands were visualized using an enhanced chemiluminescence detection system. The specific antibodies and their corresponding dilution ratios used in this study are listed in Table S5.

### 2.4 Cell Viability and LDH Release Assays

For cell viability assays, MDA‐MB‐231 and BT‐549 cells were transfected with plasmids or treated with the indicated pharmacological agents for 24 h. Cells were then trypsinized, counted, and seeded into 96‐well plates at a density of 5000 cells per well. After 48 h of treatment, cell viability was evaluated using the MTT Cell Proliferation Assay Kit (Sigma, USA) according to the manufacturer's protocol. Absorbance was recorded at 490 nm using a microplate reader.

For the lactate dehydrogenase (LDH) release assay, cells were treated identically and cultured for 48 h. Subsequently, 50 µL of the culture supernatant from each well was transferred to a fresh 96‐well plate. The LDH working solution was prepared according to the manufacturer's instructions, and 50 µL was added to each well. The plates were gently mixed and incubated at 37°C in the dark for 30 min. Absorbance was then measured at 450 nm using a microplate reader.

### 2.5 Animal Models and In Vivo Treatments

Four‐week‐old female BALB/c mice were purchased from SLAC Laboratory Animal Co., Ltd. (Shanghai, China) and randomly assigned to experimental groups (*n* = 6 per group). 4T1 cells stably expressing wild‐type (WT) ANT2 or the ANT2 K92R mutant (1 × 10^6^ cells suspended in 150 µL of serum‐free DMEM) were subcutaneously injected into the flanks of the mice. For the in vivo lactate treatment model, mice received daily intraperitoneal (i.p.) injections of NALA (120 mg/mouse) or an equivalent volume of saline. Twenty days post‐injection, mice were euthanized, and tumors were excised for downstream analyses.

For the chemo‐immunotherapy model, treatments were initiated when tumor volumes reached 50–100 mm^3^. Mice were treated with cisplatin (5 mg/kg, i.p., once weekly) and an anti‐mouse PD‐1 antibody (aPD‐1; 250 µg/mouse, BioXcell, administered every 2 days for a total of four doses). Concurrently, NALA or saline (120 mg/mouse, i.p.) was administered daily.

#### 2.5.1 Peptide Treatments

Peptides utilized in this study were synthesized and purified to > 95% purity via high‐performance liquid chromatography (HPLC) by Synpeptide Co., Ltd. (Shanghai, China). Peptides intended for in vivo use were synthesized using D‐amino acids to enhance metabolic stability. For animal administration, the P3 peptide was dissolved in PBS to prepare a working solution and stored at low temperatures. Prior to injection, the solution was equilibrated to room temperature.

### 2.6 Immunoprecipitation (Co‐IP)

Cells were lysed in Co‐IP lysis buffer supplemented with protease and phosphatase inhibitor cocktails. The lysates were centrifuged at 12 000 × *g* for 10 min at 4°C. A fraction of the resulting supernatant was collected as the input control. The remaining supernatant was incubated with specific primary antibodies on a rotator overnight at 4°C. Following incubation, 5 µL of Protein A and 5 µL of Protein G magnetic beads were added to the mixture and gently rotated for 3 h at 4°C. The bead‐protein complexes were pelleted by centrifugation at 12 000 × *g* for 1 min and washed five times with 0.5 mL of 1× Wash Buffer. The immunoprecipitated complexes were then resuspended in 20–40 µL of 1× SDS loading buffer, vortexed, and briefly centrifuged to collect the contents at the bottom of the tube. Samples were boiled at 95°C–100°C for 2–5 min, centrifuged at 14 000 × *g* for 1 min, and the supernatants were collected for immunoblotting analysis.

### 2.7 In Vitro Lactylation Assay

Recombinant His‐tagged ANT2 protein was incubated with HA‐tagged KAT8 protein (purified from HEK293T cells) in a reaction buffer containing 20 mm lactyl‐CoA. The reaction mixture was incubated at 30°C for 30 min. The reaction was terminated by the addition of SDS loading buffer and boiled at 100°C for 5 min. The samples were subsequently analyzed by immunoblotting.

### 2.8 Silver Staining and Mass Spectrometry (LC‐MS/MS)

MDA‐MB‐231 cells were transfected with a Myc‐tagged ANT2 plasmid for 48 h, followed by treatment with NALA for 3 h. Cell lysates were subjected to immunoprecipitation using an anti‐Myc epitope tag antibody. Interacting proteins and specific lactylation sites on ANT2 were subsequently identified by liquid chromatography‐tandem mass spectrometry (LC‐MS/MS) performed by Shanghai Bioprofile Technology Co., Ltd. (Shanghai, China).

### 2.9 Measurement of Intracellular Lactate, ROS, and Mitochondrial Membrane Potential

Intracellular lactate levels were quantified using a Lactate Colorimetric Assay Kit (KTB1100, Abbkine) according to the manufacturer's instructions. Mitochondrial membrane potential was assessed using a JC‐1 Assay Kit (M8650, Solarbio, China). Intracellular reactive oxygen species (ROS) levels were measured using an ROS Assay Kit (S0033S, Beyotime, China) following the manufacturer's protocols.

### 2.10 Mitochondrial DNA (mtDNA) Isolation and Quantification

Cells were divided into two equal aliquots. The first aliquot was resuspended in 300 µL of 50 mm NaOH and boiled at 95°C for 30 min to lyse the cells and release total DNA. The solution was neutralized with 30 µL of 1 m Tris‐HCl (pH 8.0). This total DNA extract served as a normalization control. The second aliquot was resuspended in 300 µL of permeabilization buffer containing 150 mm NaCl, 50 mm HEPES (pH 7.4), and 25 µg/mL digitonin (EMD Chemicals). Samples were incubated on an end‐over‐end rotator for 10 min at room temperature to selectively permeabilize the plasma membrane. The intact cells were pelleted by centrifugation at 980 × *g* for 3 min at 4°C (repeated three times). The resulting cytosolic supernatant was further centrifuged at 17 000 × *g* for 20 min to remove residual cellular debris. DNA was extracted from both the total cell lysates and the cytosolic fractions and subjected to quantitative PCR (qPCR) analysis. Nuclear DNA was amplified using primers targeting *TERT*, while mtDNA was amplified using primers for *ND5*, *D‐loop*, and *COX1*. The relative abundance of cytosolic mtDNA was calculated by normalizing the Ct values of the cytosolic fraction to those of the whole‐cell extract.

### 2.11 Fluorescence Imaging of mtDNA Leakage

Cells were seeded in glass‐bottom confocal dishes and grown to 60%–70% confluence. Prior to imaging, cells were washed once with pre‐warmed medium and incubated with MitoTracker working solution for 30 min at 37°C with 5% CO_2_ to delineate the mitochondrial network. Cells were then gently washed with fresh medium to remove unbound dye and subsequently incubated with Hoechst 33342 for 30 min at 37°C to stain the DNA. After staining, the medium was replaced with phenol red‐free medium to minimize background fluorescence. Live‐cell imaging was performed using a confocal laser scanning microscope at 37°C. Hoechst signals were utilized to visualize both nuclear and cytosolic mtDNA, while the MitoTracker signal labeled mitochondrial structures. Images were acquired using multi‐channel fluorescence settings, and the colocalization and cytosolic distribution of DNA signals were quantified utilizing ImageJ software.

### 2.12 Immunohistochemistry (IHC)

Patient tissue specimens were fixed in formalin, dehydrated through a graded ethanol series, embedded in paraffin, and sectioned. The tissue sections were incubated with specific primary antibodies against LCK, IRF1, GNLY, PDCD1, and NKG7. Following appropriate secondary antibody incubation and staining, slides were imaged under a light microscope and quantified using ImageJ software. For each section, five randomly selected high‐power fields were evaluated. The percentage of positively stained cells and the staining intensity were recorded. An IHC score was calculated by multiplying the percentage of positive cells by the staining intensity score to represent the relative protein expression levels.

### 2.13 Patient‐Derived Organoids (PDOs)

Fresh human breast cancer tissues were obtained from the Department of Thyroid and Breast Surgery, Shanghai Tenth People's Hospital. Tissues were immediately placed in pre‐cooled (2°C–8°C) Tissue Preservation Buffer E and rapidly transported to a sterile laboratory. Tissues were transferred to a petri dish, washed three times with Primary Culture Buffer B, and minced into 1–3 mm^3^ fragments using surgical scissors or a scalpel. The fragments were enzymatically dissociated in Human Breast Cancer Primary Tissue Digestion Buffer C at 37°C for 10–20 min with agitation. Digestion progress was monitored microscopically; upon observing a predominance of single cells or cell clusters smaller than 70 µm, the reaction was terminated by adding three volumes of Buffer B. The suspension was filtered through a 100‐µm cell strainer. The filtrate was centrifuged at 300 × *g* for 5 min, the supernatant was discarded, and the pellet was washed and resuspended in Buffer B. The resulting cell pellet was resuspended in Matrigel (abs9495, Absin) at a ratio of 1:25 (tissue volume to Matrigel volume). The suspension was seeded into culture plates and incubated at 37°C for 10–15 min to allow polymerization. Subsequently, Human Breast Cancer Organoid Culture Medium A (equilibrated to room temperature) was added. Once the organoids reached appropriate dimensions, they were subjected to pharmacological treatments. The morphological integrity of the organoid clusters in the treatment and control groups was assessed using bright‐field microscopy.

### 2.14 mPTP Opening Assay

Cells were seeded in 6‐well plates and incubated for 24 h at 37°C in a 5% CO_2_ incubator. Following the designated treatments (with an inducer‐free medium serving as the negative control), cells were washed twice with PBS. To assess mPTP opening, cells were co‐incubated with 1 mL of Calcein AM staining solution, fluorescence quenching solution (CoCl_2_), ionomycin control, and mitotracker for 30–45 min at 37°C in the dark. The staining mixture was then replaced with fresh, pre‐warmed culture medium, and cells were incubated for an additional 30 min at 37°C in the dark to ensure complete hydrolysis of Calcein AM by intracellular esterases, yielding green fluorescent calcein. After washing 2–3 times with PBS, Assay Buffer was added, and the fluorescence signals were visualized and captured using a fluorescence microscope.

### 2.15 Chronic Restraint Stress (CRS) Model

To establish an orthotopic breast cancer model under chronic stress, TNBC cells in the logarithmic growth phase were resuspended in PBS and injected into the fourth mammary fat pads of female mice (5 × 10^5^ cells per mouse). Seven days post‐inoculation (when tumor volumes reached approximately 50–100 mm^3^), the chronic restraint stress (CRS) protocol was initiated. Mice were placed in well‐ventilated, transparent physical restrainers (sized specifically for mice) that restricted movement but allowed normal respiration. The restraint was applied for 2 h daily for 14 consecutive days. To prevent habituation, the stress sessions were conducted at randomized times each day. Control mice were housed under identical environmental conditions but were not subjected to restraint. All mice were maintained under standard laboratory conditions (22 ± 2°C, 12‐h light/dark cycle) with ad libitum access to food and water outside of the restraint periods. At the end of the experiment, mice were euthanized, and blood and tumor tissues were collected for downstream analyses, including lactate quantification.

### 2.16 Mitochondrial Calcium Retention Capacity (CRC) Assay

The CRC assay was performed using a multimode microplate reader equipped with automated injectors. Mitochondria were isolated from WT or ANT2‐K92R mutant TNBC cells pre‐cultured with or without 50 mm NALA. Isolated mitochondria were loaded into a 96‐well black plate at a standardized concentration of 0.5 mg/mL in respiration buffer (125 mm KCl, 20 mm HEPES, 2 mm KH_2_PO_4_, 1 mm MgCl_2_, 5 mm glutamate, and 5 mm malate, pH 7.4) supplemented with 1 µm Calcium Green‐5N. Following baseline stabilization at 37°C, the system delivered sequential 20 µm CaCl_2_ pulses via automated injectors at 120‐s intervals with intermittent orbital shaking. The mPTP opening was identified by the massive and irreversible release of sequestered Ca^2+^, monitored as an abrupt increase in extramitochondrial fluorescence.

### 2.17 Isolation of Highly Purified Mitochondria

Intact mitochondria were isolated from cultured cells using the Cell Mitochondria Isolation Kit (Beyotime, C3602S) according to the manufacturer's instructions. Briefly, harvested cells were washed with ice‐cold PBS, resuspended in Mitochondrial Isolation Reagent A, and incubated on ice for 15 min. Cell rupture was achieved via Dounce homogenization, and the homogenate was centrifuged at 1200 × g for 10 min at 4°C to eliminate nuclei and unbroken cells. The resulting supernatant was subsequently centrifuged at 12 000 × g for 10 min at 4°C to precipitate the crude mitochondrial fraction. To obtain highly purified mitochondria suitable for functional assays, the crude pellet was resuspended, carefully layered over a customized gradient purification buffer (Reagent A and B mixture), and centrifuged at 21 000 × g for 10 min at 4°C. The purified mitochondrial pellet was washed once at 15 000 × g for 5 min and immediately resuspended in the respective respiration buffer for subsequent structural and functional analyses.

### 2.18 Statistical Analysis

Statistical analyses were performed using GraphPad Prism and R software. Data are presented as mean ± SD (or SEM, as indicated). Prior to significance testing, data distribution was evaluated for normality using the Shapiro‐Wilk test, and the equality of variances was assessed using the F‐test or Levene's test. For comparisons between two independent groups, an unpaired two‐tailed Student's t‐test was used for normally distributed data with equal variance; Welch's t‐test was applied when variances were unequal. For non‐normally distributed data, the Mann‐Whitney U test was utilized. For comparisons involving three or more groups, a one‐way or two‐way analysis of variance (ANOVA) was performed, followed by appropriate post hoc multiple‐comparison corrections (e.g., Tukey's or Šídák's tests). For high‐throughput omics data, *p*‐values were adjusted using the Benjamini‐Hochberg procedure to control the false discovery rate (FDR). Statistical significance was defined as *p* < 0.05. ^*^ *p* < 0.05, ^**^ *p* < 0.01, ^***^ *p* < 0.001, ^****^ *p* < 0.0001; ns, not significant.

## 3 Results

### 3.1 Tumor‐Derived Lactate Correlates With Resistance to Chemo‐Immunotherapy

While chemo‐immunotherapy is widely utilized, the specific metabolic factors changing its efficacy remain unclear. To define the metabolic factors influencing chemo‐immunotherapy efficacy, we analyzed single‐cell RNA sequencing (scRNA‐seq) profiles (GSE305078) from breast cancer patients receiving chemo‐immunotherapy [22]. Transcriptomic stratification revealed an enriched glycolysis signature specifically within the non‐responder cohort (Figure 1A). Consistently, analysis of TCGA breast cancer datasets using a Random Forest‐based immunophenoscore (IPS) algorithm showed that patients with lower expression of LDHA—the rate‐limiting enzyme for lactate production—exhibited better predictive responses to immune checkpoint blockade (Figure 1B; Table S1). To determine whether intratumoral lactate accumulation was directly associated with immune evasion, we established a paired metabolomic‐transcriptomic breast cancer cohort. These patients exhibited no significant baseline differences in age, TNM staging, or histological pathology (Table S2). Based on mass spectrometry analysis of the surgical specimens, patients were metabolically stratified into low‐ and high‐lactate groups. Notably, patients within the low‐lactate tier presented with higher overall immune activity scores (Figure 1C). Transcriptomic profiling revealed that tumors in the low‐lactate group displayed enrichment across five core immune modules: T cell activation, IFN‐γ signaling, cytotoxicity, antigen presentation, and immune checkpoint expression. These signatures were blunted in the high‐lactate group (Figure 1D; Figure S1A). These transcriptomic profiles were validated by immunohistochemistry (IHC) in patient specimens (Figure 1E,F). To investigate the impact of lactate in vivo, exogenous NALA attenuated the tumor‐suppressive effects of combined chemo‐immunotherapy (Figure 1G,H; Figure S1B). Concomitantly, immunofluorescence staining of the corresponding murine tumor tissues showed that NALA treatment restricted the intratumoral infiltration of CD8^+^ T cells (Figure 1I,J). This observation was corroborated by flow cytometry, which confirmed a decreased proportion of infiltrating CD8+ T cells in the lactate‐treated group compared to the control (Figure S1C). Together, these results suggest that lactate is associated with an immunosuppressive microenvironment and contributes to chemo‐immunotherapy resistance.

**FIGURE 1:**
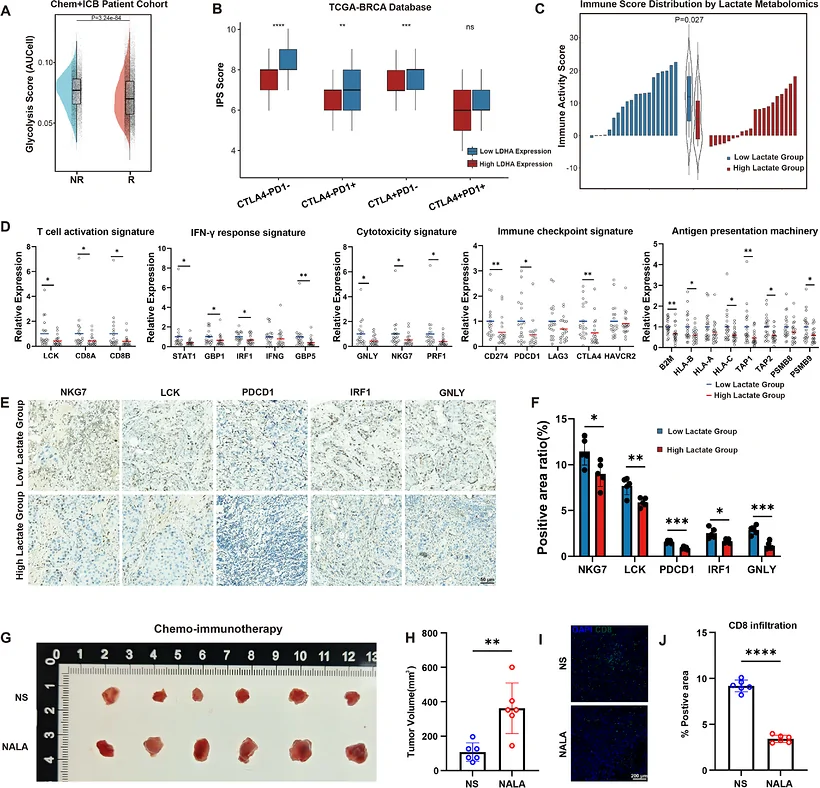
Tumor‐derived lactate drives resistance to chemo‐immunotherapy. (A) Violin plot comparing the glycolysis scores (calculated via AUCell) between non‐responders (NR) and responders (R) in a neoadjuvant chemo‐immunotherapy breast cancer patient cohort. Statistical significance was determined by the two‐sided Mann‐Whitney U test. (B) Immunophenoscore (IPS) predictions for immune checkpoint blockade efficacy based on low (blue) vs. high (red) LDHA expression in the TCGA‐BRCA dataset. Data are presented as box‐and‐whisker plots. Statistical significance was determined by the two‐sided Mann‐Whitney U test. (C) Distribution of overall immune activity scores in a clinical breast cancer cohort (*n* = 20 per group), metabolically stratified into low‐lactate (blue) and high‐lactate (red) groups. Statistical significance was determined by the two‐sided Mann‐Whitney U test. (D) Relative transcriptomic expression of key genes characterizing five core immune modules: T cell activation, IFN‐γ response, cytotoxicity, immune checkpoints, and antigen presentation machinery, compared between the low‐ and high‐lactate patient cohorts (*n* = 20 per group). Horizontal red/blue lines indicate the mean values. Statistical significance was determined by the two‐sided Mann‐Whitney U test. (E) Representative immunohistochemistry (IHC) images showing the protein expression of NKG7, LCK, PDCD1, IRF1, and GNLY in tumor sections from the low‐ and high‐lactate groups. Scale bars, 50 µm. (F) Quantitative analysis of the IHC positive area ratios for the markers shown in (E). Data are presented as mean ± SD. Statistical significance was determined by the two‐sided Mann‐Whitney U test. (G) Excised orthotopic 4T1 breast tumors from the normal saline (NS) control and NALA‐treated groups at the experimental endpoint following combined chemo‐immunotherapy (*n* = 6 mice per group). (H) Quantification of final tumor volumes corresponding to the cohorts in (G). Data are presented as mean ± SD. Statistical significance was determined by the unpaired two‐tailed Student's t‐test. (I) Representative immunofluorescence images demonstrating CD8^+^ T cell infiltration (green) and DAPI nuclear counterstaining (blue) in the murine tumor sections. Scale bars, 200 µm. (J) Quantification of the CD8^+^ positive area from the immunofluorescence images in (I). Data are presented as mean ± SD. Statistical significance was determined by the unpaired two‐tailed Student's t‐test. ^*^ *p* < 0.05, ^**^ *p* < 0.01, ^***^ *p* < 0.001, ^****^ *p* < 0.0001; ns, not significant.

### 3.2 Lactate Restricts PANoptosis to Foster an Immunosuppressive Microenvironment

Deconvolution of the scRNA‐seq cohort identified the malignant epithelial compartment as the primary source of elevated glycolysis in non‐responders (Figure 2A; Figure S1D). Single‐sample GSEA (ssGSEA) further linked high intratumoral lactate to the attenuation of programmed cell death pathways—encompassing apoptosis, necroptosis, and pyroptosis—concomitant with a suppressed interferon response (Figure 2B). To assess whether lactate directly protects cells from stress, we treated tumor cells with cisplatin in vitro. NALA administration protected tumor cells from cisplatin‐induced cytotoxicity, an effect that was reversed by the glycolysis inhibitor 2‐DG (Figure 2C–F). Similar findings were observed in patient‐derived organoids (PDOs), where lactate preserved structural integrity during chemotherapeutic challenge (Figure 2G).

**FIGURE 2:**
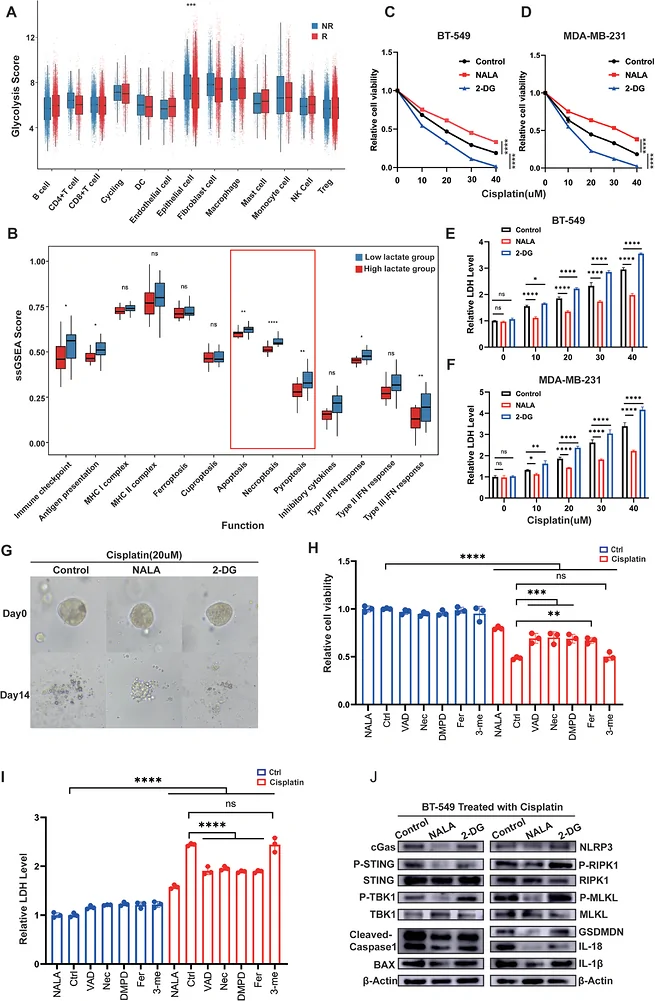
Lactate restricts tumor cell PANoptosis. (A) Box‐and‐whisker plots comparing the glycolysis scores of distinct cellular subpopulations within the tumor microenvironment of non‐responders (NR, blue) vs. responders (R, red), derived from the scRNA‐seq cohort. Statistical significance was determined by the two‐sided Mann‐Whitney U test. (B) Single‐sample gene set enrichment analysis (ssGSEA) of functional immune and cell death modules in patient cohorts stratified by low (blue) and high (red) lactate levels. The red box highlights the profound attenuation of PANoptosis‐related pathways (apoptosis, necroptosis, and pyroptosis) in the high‐lactate group. Statistical significance was determined by the two‐sided Mann‐Whitney U test. (C, D) Relative cell viability of BT‐549 (C) and MDA‐MB‐231 (D) breast cancer cells treated with the indicated concentrations of cisplatin for 48 h, in the presence of vehicle control (black), sodium lactate (NALA, red), or 2‐deoxy‐D‐glucose (2‐DG, blue). Data are presented as mean ± SEM. Statistical significance was determined by two‐way ANOVA followed by Tukey's multiple comparisons test. (E, F) Quantification of relative lactate dehydrogenase (LDH) release from BT‐549 (E) and MDA‐MB‐231 (F) cells under identical conditions to those in (C, D). Data are presented as mean ± SEM. Statistical significance was determined by two‐way ANOVA followed by Tukey's multiple comparisons test. (G) Representative bright‐field images of patient‐derived breast cancer organoids (PDOs) exposed to 20 µM cisplatin for 14 days alongside vehicle control, NALA, or 2‐DG. Lactate treatment structurally preserves the organoids against chemotherapeutic stress (*n* = 3). (H, I) Pharmacological rescue assays assessing the impact of specific cell death inhibitors on BT‐549 cells. Cells were evaluated for relative cell viability (H) and relative LDH release (I) following treatment with vehicle control (Ctrl), NALA, or specific inhibitors targeting apoptosis (Z‐VAD‐FMK [VAD]), necroptosis (necrostatin‐1 [Nec]), pyroptosis (Ac‐DMPD [DMPD]), ferroptosis (ferrostatin‐1 [Fer]), or autophagy (3‐methyladenine [3‐me]), in the absence (blue) or presence (red) of cisplatin. Data are presented as mean ± SEM. Statistical significance was determined by one‐way ANOVA followed by Dunnett's multiple comparisons test. (J) Immunoblotting analysis of PANoptosis executioners and cGAS‐STING innate DNA‐sensing pathway components in BT‐549 cells treated with cisplatin, supplemented with NALA or 2‐DG. β‐Actin served as the loading control. ^*^ *p* < 0.05, ^**^ *p* < 0.01, ^***^ *p* < 0.001, ^****^ *p* < 0.0001; ns, not significant.

We next investigated the specific cell death cascades involved in this process. Pharmacological profiling indicated that lactate confers broad‐spectrum cell death resistance rather than blocking a single cascade. Cisplatin‐induced cell death was partially rescued by independent blockade of apoptosis (Z‐VAD‐FMK), necroptosis (necrostatin‐1), pyroptosis (Ac‐DMPD), and ferroptosis (ferrostatin‐1), whereas autophagy inhibition (3‐methyladenine) exerted no effect (Figure 2H,I). Biochemical validation corroborated this pan‐inhibitory phenotype; NALA suppressed pro‐apoptotic BAX expression, prevented the phosphorylation of necroptotic executors MLKL and RIPK1, and downregulated the pyroptotic axis components NLRP3, GSDMD‐N, and Caspase‐1. Furthermore, NALA limited the activation of the cGAS‐STING innate DNA‐sensing pathway, reducing the intracellular production of pro‐inflammatory cytokines IL‐18 and IL‐1β (Figure 2J). Because the concurrent suppression of these diverse pathways is characteristic of PANoptosis, we investigated whether lactate directly influences PANoptosome assembly. The PANoptosome complex, comprising Caspase‐8, RIPK3, and ZBP1, is known to assemble in response to specific intracellular stressors [23, 24]. Using endogenous RIPK3 pull‐down assays, we observed that NALA administration markedly attenuated the assembly of the PANoptosome complex under cisplatin‐induced stress (Figure S1E). To further confirm the requirement for PANoptosis in this context, we generated Caspase‐8/ZBP1/RIPK3 triple‐knockout cells (Figure S1F). These deficient cells exhibited enhanced tolerance to cisplatin compared with wild‐type cells, and pharmacological inhibition of lactate production failed to sensitize them to chemotherapeutic stress (Figure S1G,H). Thus, our findings suggest that NALA limits the execution of tumor cell PANoptosis, restricting the immunogenic signals required to sustain an inflamed microenvironment.

### 3.3 Lactate Gates the Mitochondrial Permeability Transition Pore to Prevent mtDNA‐Driven Inflammation

Given that mitochondria regulate both cell death and intracellular inflammation [11, 25], we sought to evaluate the impact of lactate on mitochondrial dynamics under chemotherapeutic stress. We first measured reactive oxygen species (ROS) production and mitochondrial membrane potential. While NALA treatment did not significantly alter the initial cisplatin‐induced ROS burst (Figure S2A,B), it maintained the mitochondrial membrane potential (Figure S2C; Figure 3A,B). To explore how lactate preserves mitochondrial integrity, we employed the calcein‐CoCl_2_ quenching assay to monitor pore dynamics. The results indicated that lactate supplementation limited the opening of the mitochondrial permeability transition pore (mPTP) (Figure 3C,D). This mPTP‐inhibitory effect was further corroborated by a calcium retention capacity (CRC) assay. Mitochondria isolated from NALA‐treated cells withstood more repeated calcium pulses before undergoing massive pore opening compared to control mitochondria (Figure S2D). Given that prolonged mPTP opening facilitates the release of mitochondrial components, we tracked mitochondrial DNA (mtDNA) and observed that NALA reduced the cytosolic translocation of mtDNA, a well‐established trigger for intracellular inflammatory signaling (Figure 3E–G).

**FIGURE 3:**
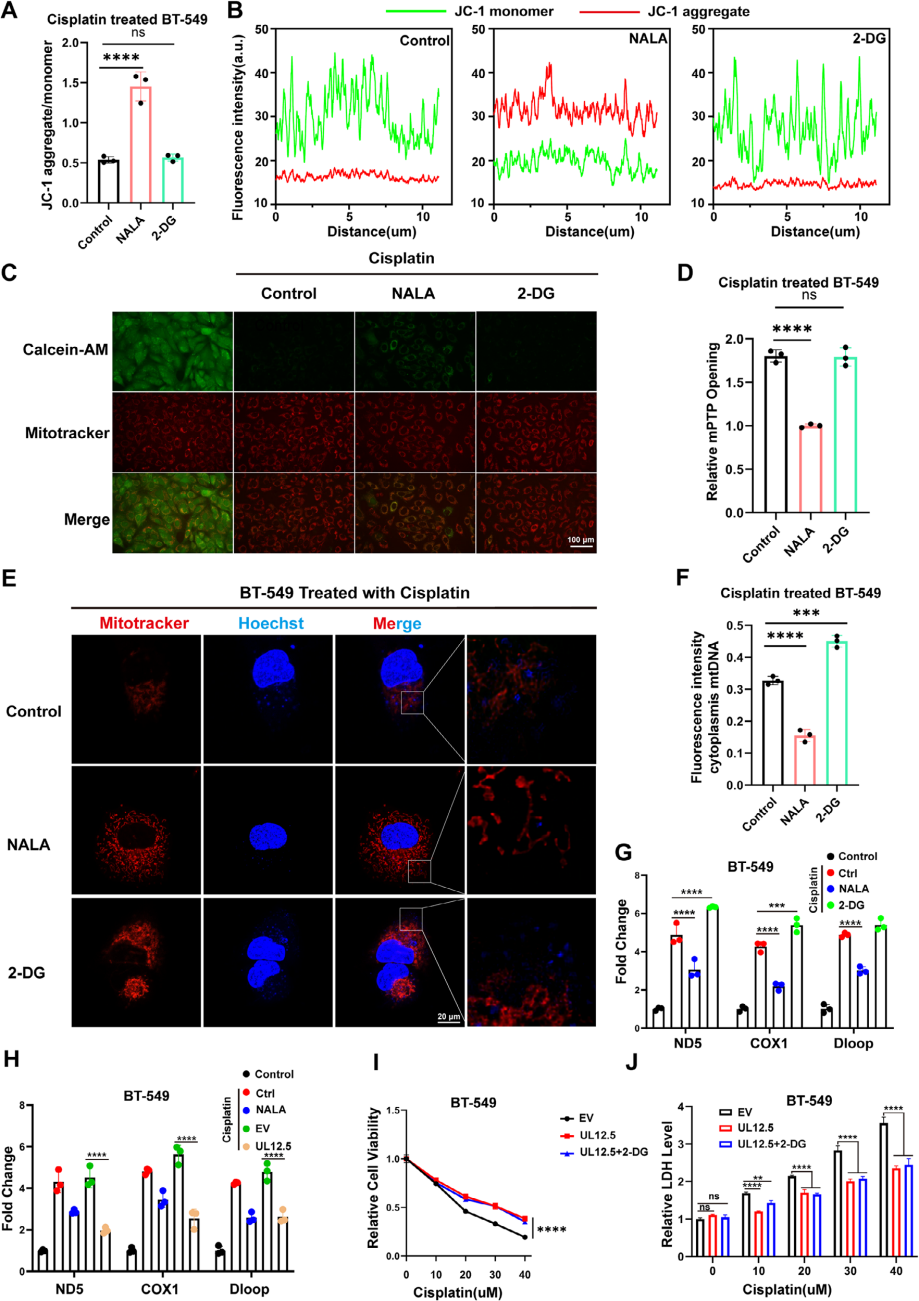
Lactate limits mPTP opening to prevent mtDNA leakage. (A) Quantitative analysis of the mitochondrial membrane potential (MMP) in cisplatin‐stressed BT‐549 cells, expressed as the ratio of JC‐1 aggregates (red) to monomers (green). Cells were co‐treated with vehicle control, NALA, or 2‐DG. Data are presented as mean ± SEM. Statistical significance was determined by one‐way ANOVA followed by Tukey's multiple comparisons test. (B) Representative spatial fluorescence intensity profiles of JC‐1 monomers and aggregates across individual cells from the indicated treatment groups in (A). (C) Representative fluorescence images of the mPTP opening assay utilizing the calcein‐CoCl_2_ quenching method. MitoTracker (red) demarcates the mitochondrial network, while retained calcein fluorescence (green) indicates mPTP closure, Scale bars, 100 µm. (D) Relative quantification of mPTP opening derived from the calcein quenching assay in (C). Data are presented as mean ± SEM. Statistical significance was determined by one‐way ANOVA followed by Tukey's multiple comparisons test. (E) Representative confocal microscopy images evaluating the cytosolic leakage of mitochondrial DNA (mtDNA). BT‐549 cells were stained with MitoTracker (red) and Hoechst 33342 (blue) to visualize mitochondria and DNA, respectively. Magnified insets highlight the presence or absence of cytosolic mtDNA signals, Scale bars, 20 µm. (F) Quantification of cytosolic mtDNA fluorescence intensity from the confocal images in (E). Data are presented as mean ± SEM. Statistical significance was determined by one‐way ANOVA followed by Tukey's multiple comparisons test. (G) Quantitative PCR (qPCR) analysis of cytosolic mtDNA abundance (*ND5*, *COX1*, and *D‐loop*), normalized to total cellular DNA, in BT‐549 cells subjected to the indicated treatments under chemotherapeutic stress. Data are presented as mean ± SEM. Statistical significance was determined by one‐way ANOVA followed by Dunnett's multiple comparisons test. (H) qPCR validation of cytosolic mtDNA depletion in cisplatin‐treated BT‐549 cells stably expressing the viral exonuclease UL12.5 or an empty vector (EV), compared to baseline treatment controls. Data are presented as mean ± SEM. Statistical significance between the EV and UL12.5 groups was determined by the unpaired two‐tailed Student's t‐test. (I, J) Relative cell viability (I) and quantification of LDH release (J) in EV‐ or UL12.5‐expressing BT‐549 cells treated with the indicated concentrations of cisplatin for 48 h, in the presence or absence of 2‐DG. Data are presented as mean ± SEM. Statistical significance was determined by two‐way ANOVA followed by Tukey's multiple comparisons test. ^*^ *p* < 0.05, ^**^ *p* < 0.01, ^***^ *p* < 0.001, ^****^ *p* < 0.0001; ns, not significant.

To further determine whether the restriction of cytosolic mtDNA is functionally responsible for lactate‐mediated chemoresistance, we engineered tumor cells to express the herpes simplex virus nuclease UL12.5. This model allows for the targeted depletion of mtDNA independent of its canonical nuclease activity, effectively uncoupling mtDNA presence from upstream mPT signaling [26, 27] (Figure 3H; Figure S2E). Notably, UL12.5‐mediated mtDNA depletion phenocopied the protective phenotype of lactate treatment, rendering cells resistant to cisplatin‐induced death. Furthermore, this resistance could not be sensitized by the glycolysis inhibitor 2‐DG (Figure 3I,J). positioning mtDNA release as an important downstream event. Collectively, these data suggest that lactate contributes to chemotherapeutic resistance by limiting mPTP opening and restricting mtDNA‐dependent cellular inflammation.

### 3.4 ANT2 Lactylation at K92 Preserves Mitochondrial Integrity Under Chemotherapeutic Stress

Lactate functions not only as a metabolic byproduct but also as a signaling molecule capable of driving lysine lactylation to regulate cellular stress responses [12, 14, 28]. Given that exogenous lactate maintained mPTP closure under chemotherapeutic stress, we hypothesized that lactate confers this mitochondrial resilience through the direct lactylation of mPTP regulatory components. To test this, we performed quantitative lactylome profiling of breast cancer cells. Among the 1,206 identified lactylated proteins, 86 were localized to the mitochondria (Table S3). By cross‐referencing this mitochondrial lactylome with the established mPTP interactome network [29], we pinpointed 20 candidate proteins. The inner mitochondrial membrane translocator ANT2 exhibited the highest lactylation stoichiometry, prompting functional investigation (Figure 4A).

**FIGURE 4:**
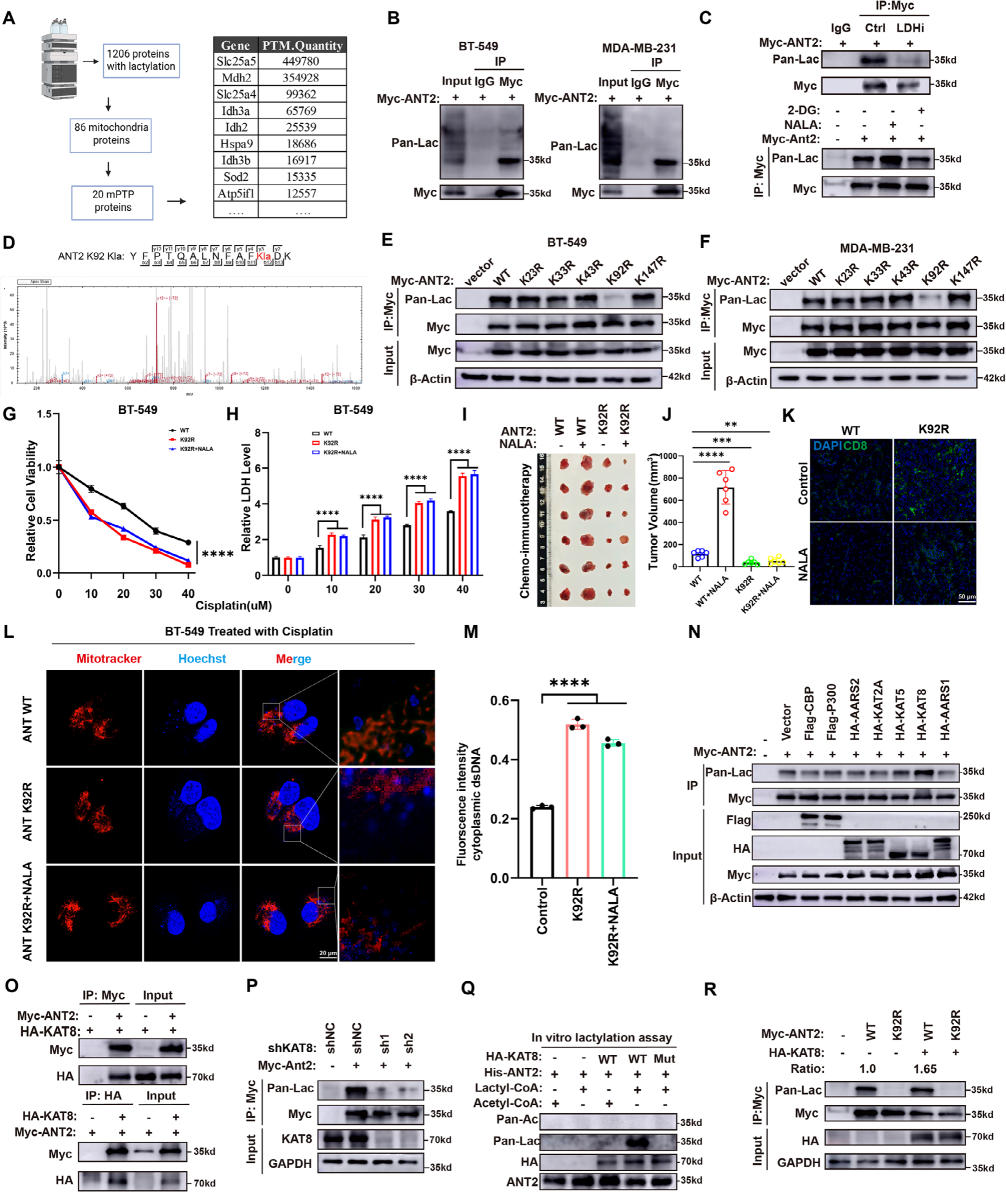
KAT8 lactylates ANT2 at K92 to maintain mitochondrial stability. (A) Schematic overview of the quantitative lactylome profiling workflow (left) and a ranked list of mPTP‐associated proteins based on their lactylation stoichiometry, identifying ANT2 (Slc25a5) as the top candidate (right). (B) Immunoprecipitation (IP) assays validating the basal lactylation of Myc‐tagged ANT2 in BT‐549 and MDA‐MB‐231 cells using a Pan‐Kla antibody. (C) Immunoblotting analysis demonstrating the dynamic regulation of ANT2 lactylation in response to exogenous NALA or the glycolysis inhibitor 2‐deoxy‐D‐glucose (2‐DG). (D) High‐resolution LC‐MS/MS spectrum of purified ANT2 protein, unambiguously mapping the lactylation modification to the lysine 92 (K92) residue. (E, F) Site‐directed mutational scanning of putative ANT2 lactylation sites via lysine‐to‐arginine (K‐to‐R) substitutions. Immunoblotting confirms K92 as the predominant lactylation site in both BT‐549 (E) and MDA‐MB‐231 (F) cells. (G, H) Relative cell viability (G) and quantification of LDH release (H) in BT‐549 cells stably expressing wild‐type (WT) or K92R‐mutant ANT2. Cells were treated with the indicated concentrations of cisplatin with or without NALA for 48 h. Data are presented as mean ± SEM. Statistical significance among the groups across multiple concentrations was determined by two‐way ANOVA followed by Tukey's multiple comparisons test. (I) Representative macroscopic images of orthotopic 4T1 murine tumors expressing WT or K92R ANT2. Mice received combined chemo‐immunotherapy with or without NALA co‐administration (*n* = 6 mice per group). (J) Quantification of final tumor volumes corresponding to the cohorts in (I) (*n* = 6 mice per group). Data are presented as mean ± SD. Statistical significance among the four experimental groups was determined by one‐way ANOVA followed by Tukey's multiple comparisons test. (K) Representative immunofluorescence images demonstrating CD8^+^ T cell infiltration (green) and DAPI nuclear counterstaining (blue) within the tumor microenvironment corresponding to (I), Scale bars, 50 µm. (L, M) Representative confocal microscopy images (L) and fluorescence intensity quantification (M) evaluating cytosolic mtDNA leakage. Extramitochondrial dsDNA signals (Hoechst, blue) outside the MitoTracker‐stained network (red) were assessed in cisplatin‐stressed BT‐549 cells expressing WT or K92R ANT2 ± NALA/2‐DG, Scale bars, 20 µm. Data are presented as mean ± SEM. Statistical significance among the three groups was determined by one‐way ANOVA followed by Tukey's multiple comparisons test. (N) Immunoblot‐based screening of candidate lysine acetyltransferases (KATs), identifying KAT8 as the primary enzymatic writer catalyzing ANT2 lactylation. (O) Reciprocal Co‐IP assay in HEK293T cells validating the physical interaction between HA‐tagged KAT8 and Myc‐tagged ANT2. (P) Immunoblotting analysis showing marked attenuation of ANT2 lactylation following shRNA‐mediated knockdown of KAT8. (Q) In vitro lactylation assay utilizing purified recombinant HA‐KAT8 and His‐ANT2 proteins, demonstrating direct enzyme‐substrate modification strictly dependent on Lactyl‐CoA. (R) Immunoblotting comparison of KAT8‐mediated lactylation on WT vs. K92R‐mutant ANT2. Densitometric ratios of Pan‐Lac relative to total Myc‐ANT2 (normalized to the WT group) are provided above the blot. ^*^ *p* < 0.05, ^**^ *p* < 0.01, ^***^ *p* < 0.001, ^****^ *p* < 0.0001; ns, not significant.

To verify the presence of ANT2 lactylation in tumor cells, we ectopically expressed Myc‐tagged ANT2. Immunoprecipitation assays revealed a basal level of ANT2 lactylation, which was enhanced following exogenous lactate supplementation. Conversely, restricting endogenous lactate synthesis—either by inhibiting upstream glycolysis with 2‐DG or by directly targeting LDHA with an inhibitor (LDHi)—attenuated this modification (Figure 4B,C).

High‐resolution LC‐MS/MS of purified ANT2 identified multiple putative Kla sites, including K23, K33, K43, K92, and K147. Site‐directed mutagenesis revealed that while K23R, K33R, K43R, and K147R substitutions retained lactylation signals, the K92R mutation abolished ANT2 lactylation, establishing K92 as the predominant modification site (Figure 4D–F). Cells stably expressing the ANT2‐K92R mutant were hypersensitive to cisplatin‐induced cytotoxicity, evidenced by decreased cell viability and elevated LDH release, and were completely refractory to lactate‐mediated rescue (Figure 4G,H).

In vivo, K92R‐mutant tumors exhibited increased sensitivity to combined chemo‐immunotherapy and bypassed the immunosuppressive effects of exogenous lactate, characterized by reduced tumor burden and restored intratumoral CD8^+^ T cell infiltration (Figure 4I–K; Figure S2F–H). Mechanistically, the K92R mutation structurally unlocked the mPTP, resulting in massive cytosolic mtDNA leakage under chemotherapeutic stress despite the presence of high lactate levels (Figure 4L,M). This loss of gating control was further directly corroborated by the calcium retention capacity (CRC) assay. Mitochondria harboring the ANT2‐K92R mutation displayed a markedly lower threshold for calcium‐induced pore opening, a vulnerability that could no longer be rescued by lactate supplementation (Figure S2I). Thus, lactate gates the mPTP by driving the K92 lactylation of ANT2, which serves as a structural determinant for mitochondrial resilience under chemotherapeutic pressure.

### 3.5 KAT8 Catalyzes the Specific Lactylation of ANT2

To identify the enzymatic writer responsible for ANT2 lactylation [30], we screened seven putative lactyltransferases. Ectopic expression of KAT8, but not other candidates, increased ANT2 Kla levels (Figure 4N). Reciprocal co‐immunoprecipitation (Co‐IP) confirmed a physical interaction between ANT2 and KAT8 (Figure 4O). Consistently, KAT8 knockdown reduced basal ANT2 lactylation (Figure 4P).

Given the localization of ANT2 at the inner mitochondrial membrane (IMM), we next examined whether KAT8 physically accesses this subcellular compartment. Subcellular fractionation confirmed the presence of KAT8 within mitochondria, establishing the spatial prerequisite for this enzyme‐substrate interaction. Notably, chemotherapeutic stress did not significantly alter the subcellular distribution of KAT8, implying that stress‐induced lactylation is driven by metabolic substrate availability rather than enzyme translocation (Figure S3A). To determine whether this modification is direct, we performed in vitro reconstitution assays using purified recombinant proteins. Wild‐type KAT8, but not a catalytically inactive KAT8 mutant, directly catalyzed ANT2 lactylation in the presence of lactyl‐CoA. Because KAT8 is canonically recognized as an acetyltransferase, we concurrently evaluated its potential to acetylate ANT2. Interestingly, the addition of acetyl‐CoA to the reaction mixture yielded no detectable ANT2 acetylation, indicating that KAT8 functions as a lactyltransferase, rather than an acetyltransferase, toward ANT2 (Figure 4Q). Finally, while KAT8 overexpression augmented the lactylation of wild‐type ANT2, it completely failed to modify the ANT2‐K92R mutant (Figure 4R). Collectively, these data establish KAT8 as the direct and specific enzymatic writer responsible for ANT2 lactylation at the K92 residue.

### 3.6 Lactylated ANT2 Controls the Allosteric Dephosphorylation of the mPTP Gatekeeper CypD

To understand how ANT2 lactylation restricts mPTP gating, we focused on Cyclophilin D (CypD), a critical structural interactor of ANT2 that facilitates pore opening [31, 32], Co‐IP analysis confirmed a basal ANT2‐CypD interaction (Figure 5A). However, neither exogenous lactate nor 2‐DG altered the amount of CypD co‐precipitated with ANT2, indicating that lactate treatment does not disrupt the physical assembly of the ANT2‐CypD complex (Figure 5B). Given that the structural integrity of the ANT2‐CypD complex was maintained, we reasoned that the inhibitory signal must be relayed biochemically. Because the gating sensitivity of CypD to oxidative stress is intrinsically governed by its post‐translational modifications [33, 34, 35], we profiled the PTM status of CypD under chemotherapeutic stress. While we did not detect direct lactylation on CypD, immunoblotting revealed a marked decrease in CypD phosphorylation following lactate supplementation, an effect that was readily reversed by 2‐DG (Figure 5C). Mutational mapping based on previously reported sites [36] confirmed Ser191 as the dominant phosphorylation site (Figure 5D). To definitively establish the functional consequence of this dephosphorylation, we generated cells expressing site‐specific CypD mutants. Cells expressing the phospho‐deficient CypD‐S191A mutant exhibited robust resistance to cisplatin‐induced PANoptosis, mimicking the protective effects of lactate, and were insensitive to 2‐DG sensitization (Figure 5E,F; Figure S3B). Conversely, cells expressing the phospho‐mimetic CypD‐S191D mutant displayed enhanced sensitivity to cisplatin, which could no longer be rescued by NALA (Figure S3C,D).

**FIGURE 5:**
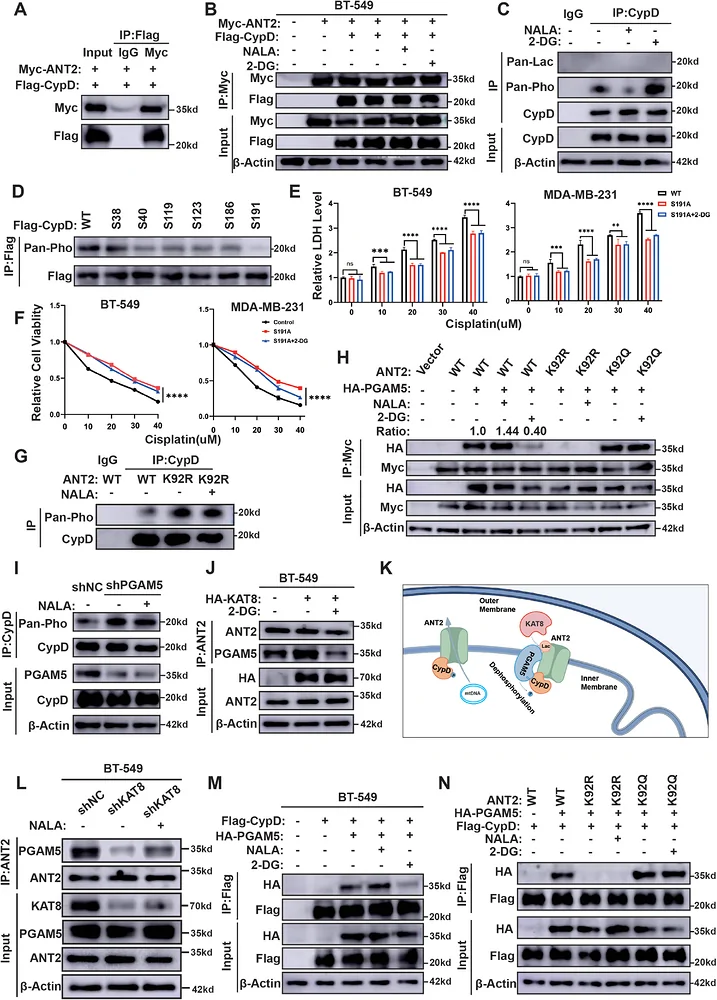
Lactylated ANT2 scaffolds PGAM5 to dephosphorylate CypD and restrict the mPT. (A) Co‐immunoprecipitation (Co‐IP) assay confirming the basal physical interaction between ectopically expressed Myc‐ANT2 and Flag‐CypD in HEK293T cells. (B) Co‐IP assay in BT‐549 cells demonstrating that the ANT2‐CypD interaction remains unaltered upon treatment with NALA or 2‐deoxy‐D‐glucose (2‐DG). (C) Immunoprecipitation of endogenous CypD followed by immunoblotting with pan‐lactylation (Pan‐Lac) and pan‐phosphoserine/threonine (Pan‐Pho) antibodies. NALA treatment profoundly reduces CypD phosphorylation but does not induce its lactylation. (D) Mutational mapping of potential phosphorylation sites on Flag‐CypD via site‐directed mutagenesis, identifying S191 as the primary phosphorylated residue. (E, F) Quantification of relative LDH release (E) and cell viability (F) in BT‐549 and MDA‐MB‐231 cells expressing wild‐type (WT) or the phospho‐ablative S191A‐mutant CypD. Cells were treated with the indicated concentrations of cisplatin with or without 2‐DG for 48 h. Data are presented as mean ± SEM. Statistical significance among the three groups across multiple concentrations was determined by two‐way ANOVA followed by Tukey's multiple comparisons test. (G) Immunoblotting analysis of endogenous CypD phosphorylation in BT‐549 cells stably expressing WT or K92R‐mutant ANT2. The K92R mutation results in constitutive CypD hyperphosphorylation that is refractory to NALA treatment. (H) Co‐IP assay evaluating the interaction between Myc‐ANT2 mutants (WT, K92R, K92Q) and HA‐PGAM5. The binding is enhanced by NALA, strictly dependent on K92 lactylation (abolished in K92R), and constitutively locked in the K92Q lactylation‐mimetic mutant. (I) Immunoblotting showing that shRNA‐mediated knockdown of *PGAM5* elevates CypD phosphorylation levels, overriding the suppressive effect of NALA. (J, L) Co‐IP assays in BT‐549 cells demonstrating that the physical interaction between ANT2 and PGAM5 is augmented by KAT8 overexpression (J) and severely impaired by KAT8 knockdown (L). (K) Proposed working model illustrating the “metabolic lock” mechanism: KAT8 lactylates ANT2 at K92, which structurally scaffolds the phosphatase PGAM5 to dephosphorylate CypD, thereby restricting mPTP opening and preserving mitochondrial integrity. (M, N) Co‐IP assays revealing the dynamic interaction between PGAM5 and CypD. This association is potentiated by NALA and disrupted by 2‐DG (M), and strictly governed by the K92 lactylation status of ANT2 (N). ^*^ *p* < 0.05, ^**^ *p* < 0.01, ^***^ *p* < 0.001, ^****^ *p* < 0.0001; ns, not significant.

Finally, we sought to verify whether this dephosphorylation event was directly downstream of the KAT8/ANT2 lactylation axis. Indeed, compared to wild‐type cells, ANT2‐K92R mutant cells exhibited inherently hyperphosphorylated CypD (Figure 5G). A similar hyperphosphorylation phenotype was observed when endogenous lactate production was blocked using an LDHA inhibitor (LDHi) (Figure S3E). Consistent with these findings, shRNA‐mediated depletion of KAT8 elevated CypD phosphorylation (Figure S3F), whereas KAT8 overexpression suppressed it (Figure S3G). Collectively, these experimental lines demonstrate that KAT8‐mediated ANT2 lactylation at K92 restricts mPTP opening by driving the subsequent dephosphorylation of CypD at Ser191.

### 3.7 Lactylated ANT2 Scaffolds PGAM5 to Dephosphorylate CypD

Since ANT2 lacks intrinsic phosphatase activity, its regulation of CypD dephosphorylation suggests the involvement of an intermediate phosphatase. To identify this effector, we analyzed the ANT2 interactome via LC‐MS/MS. Among the 13 mitochondria‐localized phosphorylation regulators identified (Table S4), we focused on PGAM5. As an inner mitochondrial membrane (IMM)‐resident phosphatase, PGAM5 colocalizes with both ANT2 and CypD. This spatial arrangement, alongside its known role in mitochondrial homeostasis [37, 38], highlighted PGAM5 as the primary candidate.

We next examined whether the assembly of ANT2 and PGAM5 is responsive to metabolic stress. Co‐immunoprecipitation (Co‐IP) assays revealed a stress‐induced interaction between ANT2 and PGAM5 (Figure S3H). Crucially, this physical association was markedly enhanced by exogenous lactate supplementation and attenuated by glycolysis inhibition via 2‐DG. This lactate‐dependency was further corroborated by blocking endogenous lactate production with an LDHA inhibitor (LDHi), which similarly abolished the ANT2‐PGAM5 interaction (Figure S3I). To determine if this recruitment is directly governed by ANT2 lactylation at K92, we utilized site‐directed mutants. The lactylation‐deficient ANT2‐K92R mutant failed to bind PGAM5, whereas the lactylation‐mimetic K92Q mutant maintained a constitutive, robust interaction that was entirely refractory to 2‐DG treatment (Figure 5H). These interaction dynamics were further confirmed in vitro via surface plasmon resonance (SPR) assays using purified ANT2 and PGAM5 proteins under corresponding conditions (Figure S3J). Molecular docking simulations supported these experimental observations by predicting a structural interface where the lactylated K92 residue of ANT2 directly engages PGAM5 (Figure S3K). Functionally, depletion of PGAM5 resulted in CypD hyperphosphorylation that could not be rescued by lactate treatment (Figure 5I), confirming PGAM5 as the indispensable downstream effector.

Finally, we traced this signaling axis back to its upstream enzymatic writer, KAT8. Overexpression of KAT8 significantly enhanced the assembly of the ANT2‐PGAM5 complex in a 2‐DG‐sensitive manner (Figure 5J,K), whereas shRNA‐mediated knockdown of KAT8 dismantled the complex (Figure 5L). Consequently, the interaction between the effector (PGAM5) and the target (CypD) precisely mirrored the dynamics of the ANT2‐PGAM5 axis: it was augmented by lactate, abrogated by 2‐DG or the ANT2‐K92R mutation, and constitutively engaged in ANT2‐K92Q expressing cells (Figure 5M,N). To functionally validate this epistatic hierarchy, we evaluated cell survival across targeted genetic rescue models. While shRNA‐mediated knockdown of KAT8 sensitized wild‐type cells to cisplatin, it completely failed to reverse the chemoresistant phenotype of ANT2‐K92Q mutant cells (Figure S4A,B), genetically confirming that KAT8 operates strictly upstream of ANT2 K92 lactylation. Consistently, silencing the downstream effector PGAM5 abolished the protective effects of exogenous lactate. Furthermore, KAT8 overexpression failed to rescue cisplatin‐induced cell death in PGAM5‐depleted cells (Figure S4C,D), demonstrating that PGAM5 is physically indispensable for KAT8‐mediated chemoresistance. Together, these stepwise mechanistic data suggest a model wherein lactylated ANT2 functions as a metabolic sensor and conformational scaffold, recruiting PGAM5 to the mPTP complex to execute CypD dephosphorylation and lock the pore in a closed state.

### 3.8 Chronic Stress Exacerbates Therapeutic Resistance, and Targeting ANT2 Lactylation Restores Chemo‐Immunotherapy Efficacy

Psychological stress is increasingly recognized as a potent suppressor of anti‐tumor immunity [39, 40]. To explore this correlation clinically, we stratified our patient cohort using the Perceived Stress Scale (PSS‐10) (Figure S4E). We observed that highly stressed patients exhibited elevated serum lactate levels, which coincided with a reduction in systemic cGAMP—a surrogate marker for innate immune activation (Figure 6A–C). Consistent with this metabolic shift, global lactylation was enriched in tumor tissues from the high‐stress cohort (Figure 6D). This systemic phenotype was reliably recapitulated in a murine chronic restraint stress (CRS) model, where stressed mice displayed increased circulating lactate alongside blunted cGAMP and IFN‐γ production (Figure 6E–H). Given that stress hormones can modulate cellular metabolism, we hypothesized that this stress‐induced lactylation is driven by accelerated glycolysis. Supporting this, in vitro treatment of tumor cells with the stress neurotransmitter norepinephrine (NE) upregulated the expression of the lactate‐producing enzyme LDHA (Figure S4F).

**FIGURE 6:**
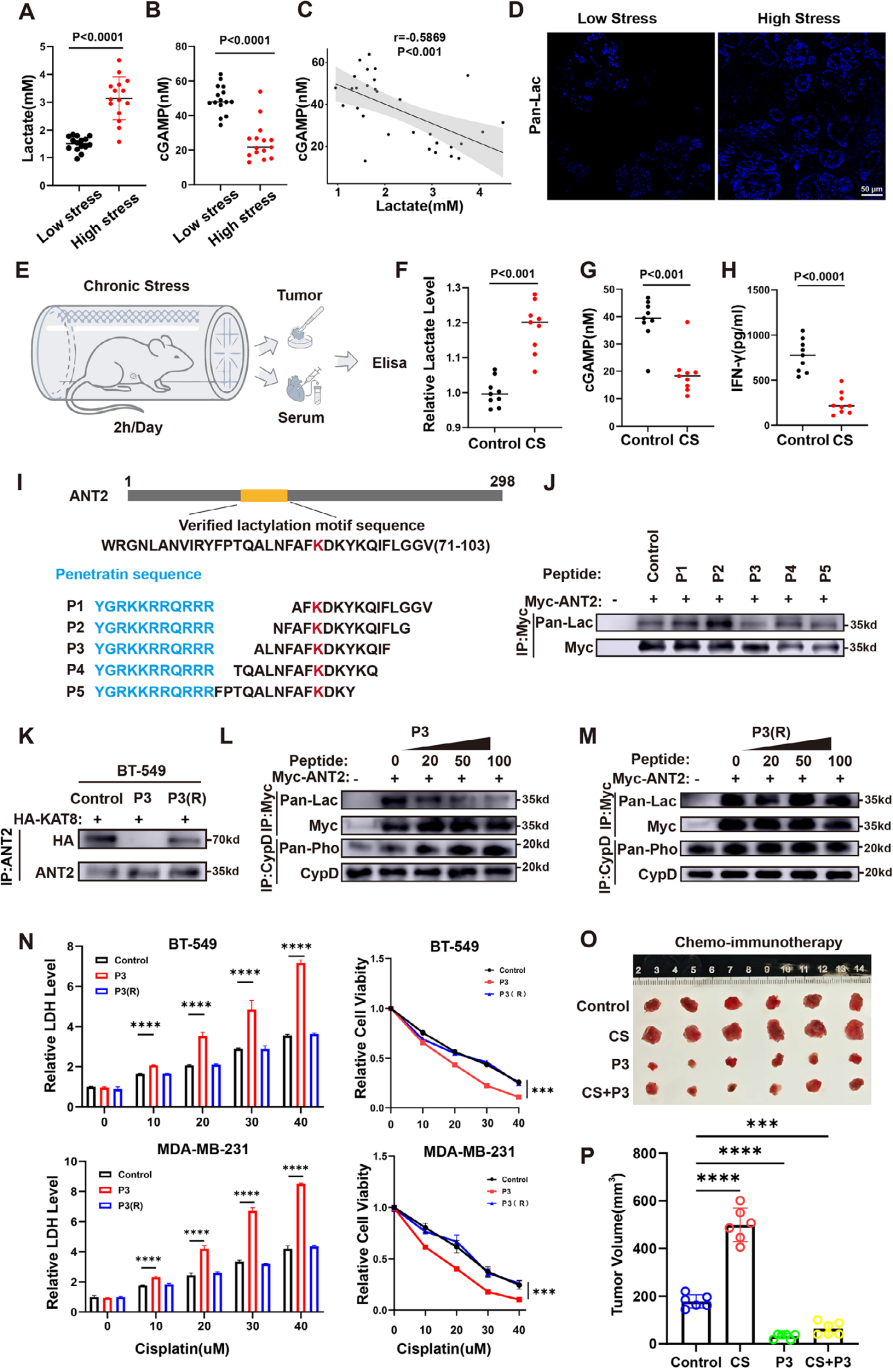
Exacerbation of resistance by chronic stress and therapeutic rescue via an ANT2‐targeted peptide. (A, B) Concentrations of serum lactate (A) and cGAMP (B) in a clinical cohort of breast cancer patients, stratified into low‐ and high‐stress groups based on the Perceived Stress Scale (PSS‐10). Data are presented as mean ± SD. Statistical significance was determined by the unpaired two‐tailed Student's t‐test. (C) Pearson correlation analysis reveals an inverse relationship between serum lactate and cGAMP levels in the clinical patient cohort. (D) Representative immunofluorescence images evaluating global protein lactylation (Pan‐Lac, blue) in tumor tissue sections from low‐ and high‐stress patients. Scale bars, 50 µm. (E) Schematic illustration of the murine chronic restraint stress (CRS) model. (F–H) Quantification of relative serum lactate (F), cGAMP (G), and IFN‐γ (H) levels in mice subjected to control conditions or the CRS protocol (*n* = 9). Data are presented as mean ± SEM. Statistical significance was determined by the unpaired two‐tailed Student's t‐test. (I) Design strategy for the cell‐penetrating competitive peptides targeting the ANT2 lactylation motif. The penetratin sequence (blue) was fused to various truncated ANT2 peptide sequences encompassing the K92 residue (red). (J) Immunoblot‐based screening of the synthesized candidate peptides (P1–P5), evaluating their capacity to competitively disrupt Myc‐ANT2 lactylation. (K) Co‐immunoprecipitation (Co‐IP) assay confirming that the active P3 peptide effectively disrupts the physical interaction between KAT8 and ANT2 in BT‐549 cells, whereas the mutant peptide P3(R) does not. (L, M) Dose‐dependent biochemical evaluation of the active P3 peptide (L) and the site‐directed mutant control peptide P3(R) (M). Ectopically expressed Myc‐ANT2 and endogenous CypD were immunoprecipitated to assess ANT2 lactylation (Pan‐Lac) and reciprocal CypD phosphorylation (Pan‐Pho) levels, respectively. (N) Quantification of relative LDH release (left panels) and relative cell viability (right panels) in BT‐549 and MDA‐MB‐231 cells. Cells were treated with the indicated concentrations of cisplatin for 48 h, in the presence of vehicle control, P3, or the P3(R) mutant peptide. Data are presented as mean ± SEM. Statistical significance among the three groups across multiple concentrations was determined by two‐way ANOVA followed by Tukey's multiple comparisons test. (O) Representative macroscopic images of orthotopic 4T1 tumors. All mice received baseline chemo‐immunotherapy and were allocated into control or CRS groups, with or without systemic administration of the P3 peptide (*n* = 6). (P) Quantification of final tumor volumes corresponding to the cohorts in (O). Data are presented as mean ± SD. Statistical significance among the four experimental groups was determined by one‐way ANOVA followed by Tukey's multiple comparisons test. ^*^ *p* < 0.05, ^**^ *p* < 0.01, ^***^ *p* < 0.001, ^****^ *p* < 0.0001; ns, not significant.

To therapeutically uncouple stress‐induced metabolism from immune evasion, we designed a cell‐penetrating competitive peptide (P3) to physically occlude the KAT8‐binding interface on ANT2 (Figure 6I,J) [41, 42]. Co‐immunoprecipitation confirmed that P3 effectively disrupted the physical interaction between KAT8 and ANT2 (Figure 6K). Consequently, P3 treatment dose‐dependently attenuated ANT2 lactylation, restored CypD phosphorylation, and sensitized TNBC cells to cisplatin. Importantly, P3 treatment did not alter global cellular lactylation levels (Figure S4G), underscoring its target specificity. A site‐directed mutant peptide P3(R) failed to exert these biochemical and functional effects, confirming sequence‐specific activity (Figure 6L,M). Finally, we evaluated the therapeutic potential of P3 in vivo. The CRS protocol severely compromised the baseline efficacy of chemo‐immunotherapy. However, systemic administration of the P3 peptide not only augmented therapeutic responses in non‐stressed mice but also effectively overcame the immunosuppressive barrier in the CRS cohort (Figure 6N–P). Profiling of the tumor microenvironment via flow cytometry and immunofluorescence revealed that chronic stress restricted CD8^+^ T cell infiltration. P3 treatment successfully reversed this immune exclusion, driving robust CD8^+^ T cell accumulation even under chronic stress conditions (Figure S4H–J). Furthermore, histological evaluation of major organs (heart, liver, spleen, lung, kidney) revealed no obvious systemic toxicity associated with P3 administration (Figure S4K). Together, these preclinical data identify the KAT8‐ANT2‐CypD axis as a potential therapeutic target to improve chemo‐immunotherapy efficacy in metabolism and stress‐induced immune resistance.

## 4 Discussion

Historically, lactate was relegated to the status of a mere metabolic byproduct of aerobic glycolysis, a passive signature of the Warburg effect. However, a major paradigm shift has repositioned intracellular lactate as a fundamental signaling hub and an epigenetic modulator of cell fate. Beyond receptor‐mediated signaling [43], lactate directly couples metabolic flux to cellular responses via lactylation (Kla), governing diverse non‐metabolic processes ranging from homologous recombination repair [14], and mitophagy [44], to innate immune surveillance [28]. While the overarching immunosuppressive role of lactate within the tumor microenvironment (TME) is well documented [45, 46], its precise intracellular function—particularly how it equips tumors to withstand the extreme stress of combined chemo‐immunotherapy—has remained a blind spot.

Chemo‐immunotherapy has emerged as a cornerstone of modern oncology, exhibiting remarkable potential across diverse malignancies [47, 48, 49, 50]. The conceptual backbone of this regimen relies on the ability of cytotoxic agents to trigger inflammatory cell death pathways, such as PANoptosis, in tumor cells. This process can release abundant damage‐associated molecular patterns (DAMPs) that potentially stimulate immune surveillance [51]. The irreversible execution of PANoptosis hinges on a catastrophic failure of mitochondrial homeostasis. Under acute chemotherapeutic stress, the mitochondrial permeability transition pore (mPTP) opens, driven by the regulatory trans‐activator Cyclophilin D (CypD), inducing a conformational shift in the inner membrane transporter ANT2. This transition allows the indiscriminate passage of molecules up to 1.5 kDa, causing rapid calcium efflux, depolarization of the mitochondrial membrane potential, and the massive cytosolic expulsion of pro‐inflammatory DAMPs (e.g., mtDNA and cytochrome *c*) [29, 52]. This explosive release is the requisite trigger for broad‐spectrum intracellular inflammation and subsequent immune activation.

Despite this robust theoretical framework, a substantial proportion of patients with triple‐negative breast cancer (TNBC), as well as those experiencing severe psychological stress [53], remain refractory to chemo‐immunotherapy, presenting an urgent and unmet clinical challenge. By integrating single‐cell transcriptomics with paired metabolomic‐transcriptomic profiling of a patient cohort, we identified the oncometabolite lactate as the dominant metabolic determinant dictating this therapeutic resistance. Crucially, our findings suggest that lactate may act as an intracellular “metabolic lock” that safeguards mitochondrial integrity against PANoptosis. Through unbiased lactylome profiling, we observed that lactate promotes the specific lactylation of ANT2 at K92. This post‐translational modification converts ANT2 into a conformational scaffold that recruits the inner membrane phosphatase PGAM5. PGAM5, in turn, dephosphorylates CypD, thereby raising the activation threshold of the mPTP and restricting the execution of PANoptosis. Furthermore, we designed a cell‐penetrating competitive peptide that precisely disrupts this ANT2 lactylation event. By biochemically unlocking the mPTP, this peptide reinstates the chemosensitivity of tumor cells and revitalizes the inflamed TME.

In a broader biological context, our study proposes a potential mechanism by which tumor cells might exploit their own metabolic byproducts to establish a structural defense against severe stress. By coupling high glycolytic flux to the epigenetic fortification of mitochondrial pores, tumors establish a resilient equilibrium that allows them to endure genotoxic insults and evade immune detection. Moreover, the hyperactivation of this lactylation axis in chronically stressed patients underscores the intricate crosstalk between systemic physiological states and localized tumor metabolism. ANT2 K92 lactylation may not only serve as a potential biomarker for predicting chemo‐immunotherapy responsiveness but also indicate a potentially targetable metabolic vulnerability. Precision intervention of this metabolic checkpoint represents a promising strategy that could help mitigate intrinsic and stress‐induced resistance, potentially expanding the clinical utility of chemo‐immunotherapy.

## 5 Limitations

Despite these mechanistic advances, certain limitations in our current study highlight critical avenues for future investigation. First, while we have established the lactate‐driven “metabolic lock” on the mPTP within the specific context of triple‐negative breast cancer (TNBC), hyperglycolysis is a ubiquitous hallmark shared by numerous solid tumors. Whether this ANT2 lactylation‐dependent mechanism universally governs chemo‐immunotherapy resistance across other highly metabolic malignancies remains a compelling question to be addressed in broader clinical cohorts. Second, from a translational perspective, although the cell‐penetrating P3 peptide provides a robust in vivo proof‐of‐concept for disarming this metabolic checkpoint, peptide‐based therapeutics inherently face pharmacological hurdles, such as limited in vivo half‐life, proteolytic degradation, and potential off‐target tissue distribution. To advance this strategy toward clinical application, future efforts must focus on structural optimization, the development of highly specific small‐molecule competitive inhibitors, or the implementation of tumor‐targeted nanocarrier delivery systems to precisely disrupt the KAT8‐ANT2 interface.

## Author Contributions

**Sen Zhong**: Writing – review & editing, Project administration, Methodology, Investigation. **Wenlong Chen**: Writing – review & editing, Validation, Investigation. **Fanglong Liu**: Methodology, Investigation. **Shengyi Zhou**: Investigation, Data curation, Conceptualization. **Bolin Yu**: Project administration, Investigation, Conceptualization. **Yuying Wang**: Software, Formal analysis. **Xiqian Zhou**: Validation, Software, Data curation. **Xuehui Wang**: Visualization. **Diya Liu**: Writing – review & editing, Writing – original draft, Data curation. **Zhuoyu Zhang**: Formal analysis. **Xinran Wang**: Validation. **Mounia Lalouly**: Project administration. **Yiwen Li**: Visualization. **Zhefei Du**: Software. **Tao Yan**: Software. **Zhihui Xiao**: Methodology. **Zhou Zhou**: Formal analysis. **Huanhuan Zhu**: Resources, **Fengyuan Qian**: Writing – review & editing, Resources, Project administration, Conceptualization. **Bowen Zheng**: Writing – review & editing, Project administration, Data curation, Conceptualization. **Lin Fang**: Writing – review & editing, Visualization, Supervision, Resources, Project administration, Funding acquisition, Formal analysis, Data curation, Conceptualization.

## Funding

This work was supported by the National Natural Science Foundation of China Grants (Grant numbers 82073204 and 82473088).

## Conflicts of Interest

The authors declare no conflicts of interest.

## Supporting information




**Supporting File 1**: advs76321‐sup‐0001‐SuppMat.docx.

**Supporting File 2**: advs76321‐sup‐0002‐TableS1‐S5.zip.

## Data Availability

The data that support the findings of this study are available in the supplementary material of this article.
