# Supplementary material for: Lactate‐Driven Restriction of Mitochondrial Permeability Transition Promotes Resistance to Chemo‐Immunotherapy by Suppressing Tumor PANoptosis
Source: Adv Sci (Weinh). 2026 Jun 30;13(53):e76321. doi: 10.1002/advs.76321 (PMC13335109; doi:10.1002/advs.76321)
Supplement: Supplementary file 1 [file ADVS-13-e76321-s002.docx]

**FigureS1.** **(A):** The heatmap of transcriptomic expression of key genes characterizing five core immune modules: T cell activation, IFN-γ response, cytotoxicity, immune checkpoints, and antigen presentation machinery, compared between the low- and high-lactate patient cohorts. **(B)** Schematic illustration of the *in vivo* treatment regimen. Mice bearing 4T1 tumors were treated with chemotherapy (Chem) and anti-PD-1, combined with normal saline (NS) or sodium lactate (NALA). **(C)** Representative flow cytometry contour plots and corresponding quantitative analysis of CD8^+^ T cell infiltration (% of CD8^+^CD45^+^ cells) in the tumors from the NS and NALA treatment groups. Data are presented as mean ± SD. Statistical significance was determined by the unpaired two-tailed Student’s t-test. **(D)** T-SNE (t-distributed stochastic neighbor embedding) visualization of the distinct cellular subpopulations identified in the single-cell RNA sequencing (scRNA-seq) cohort. **(E)** Co-immunoprecipitation (Co-IP) assay evaluating the assembly of the PANoptosome complex (interactions among RIPK3, Caspase-8, and ZBP1 with NLRP3) in WT BT-549 cells under cisplatin stress, with or without NALA supplementation. **(F)** Immunoblotting analysis confirming the successful genetic knockout of RIPK3, Caspase-8, and ZBP1 in the BT-549 triple-knockout (TKO) cell line. β-Actin served as the loading control. **(G, H)** Relative cell viability (G) and relative LDH release (H) of WT, TKO, and LDHi-treated TKO BT-549 cells exposed to the indicated concentrations of cisplatin for 48 h. Data are presented as mean ± SEM. Statistical significance among the three groups across multiple concentrations was determined by two-way ANOVA followed by Tukey's multiple comparisons test. *** P < 0.05, ** P < 0.01, *** P < 0.001, **** P < 0.0001; ns, not significant.**

**FigureS2.** **(A)** Representative fluorescence images measuring reactive oxygen species (ROS) production in cisplatin-treated BT-549 cells co-treated with vehicle control, NALA, or 2-DG. Scale bars, 100 μm. **(B)** Quantification of mean fluorescent intensity of ROS from the images in (A). Data are presented as mean ± SEM. Statistical significance was determined by one-way ANOVA followed by Tukey's multiple comparisons test. **(C)** Representative fluorescence images of the mitochondrial membrane potential (assessed via JC-1 staining) in cisplatin-treated BT-549 cells supplemented with vehicle control (Ctrl), NALA, or 2-DG. Scale bars, 100 μm. **(D)** Calcium retention capacity (CRC) assay evaluating mPTP opening thresholds in mitochondria isolated from control and lactate-treated cells. Black arrowheads indicate the sequential addition of calcium pulses. **(E)** Quantitative PCR (qPCR) validation of cytosolic mtDNA depletion (ND5, Cox1, Dloop) and the nuclear DNA control (Tert) in BT-549 cells stably expressing the empty vector (EV) or the viral exonuclease UL12.5. Data are presented as mean ± SEM. Statistical significance between EV and UL12.5 groups for each gene was determined by the unpaired two-tailed Student’s t-test. **(F)** Representative flow cytometry contour plots assessing CD8^+^ T cell infiltration in orthotopic 4T1 tumors from WT and ANT2-K92R mutant mice, treated with or without NALA. **(G, H)** Quantitative analysis of CD8^+^ T cell infiltration (% of CD8^+^CD45^+^ cells) from the flow cytometry data (G), and quantification of the CD8+ positive area from immunofluorescence staining (H) across the indicated groups. Data are presented as mean ± SD. Statistical significance among the four experimental groups was determined by one-way ANOVA followed by Tukey's multiple comparisons test. **(I)** Calcium retention capacity (CRC) assay in mitochondria isolated from WT and ANT2-K92R mutant cells, in the presence or absence of lactate treatment, demonstrating that the K92R mutation abolishes lactate-mediated mPTP inhibition. *** P < 0.05, ** P < 0.01, *** P < 0.001, **** P < 0.0001; ns, not significant.**

**Figure S3. (A)** Immunoblotting analysis of KAT8 protein levels in the cytosolic (Cyto) and mitochondrial (Mito) fractions of BT-549 cells, with or without cisplatin treatment. VDAC2 and GAPDH were used as mitochondrial and cytosolic loading controls, respectively. **(B)** Immunoblotting analysis of the cGAS-STING innate DNA-sensing pathway and PANoptosis executioners in BT-549 cells expressing wild-type (WT) or the phospho-ablative S191A-mutant CypD, treated with cisplatin alone or in combination with 2-DG. β-Actin served as the loading control. **(C, D)** Relative cell viability (C) and quantification of relative LDH release (D) in BT-549 cells expressing WT or the phospho-mimetic S191D-mutant CypD. Cells were treated with the indicated concentrations of cisplatin with or without sodium lactate (NALA) for 48 h. Data are presented as mean ± SEM. Statistical significance among the three groups across multiple concentrations was determined by two-way ANOVA followed by Tukey's multiple comparisons test. **(E)** Immunoprecipitation of endogenous CypD followed by immunoblotting with a pan-phosphoserine/threonine (Pan-Pho) antibody to evaluate CypD phosphorylation levels in BT-549 cells treated with or without an LDHA inhibitor (LDHi). **(F, G)** Immunoblotting analysis of CypD phosphorylation (Pan-Pho) in BT-549 cells subjected to shRNA-mediated KAT8 knockdown ± NALA (F), or KAT8 overexpression ± 2-DG (G). **(H)** Co-immunoprecipitation (Co-IP) assay confirming the physical interaction between Myc-ANT2 and HA-PGAM5. **(I)** Co-IP assay demonstrating that blocking endogenous lactate production with LDHi abrogates the physical interaction between Myc-ANT2 and endogenous PGAM5. **(J)** Surface plasmon resonance (SPR) assay validating the direct *in vitro* binding affinity between purified ANT2 (WT or K92R) and PGAM5 proteins, with or without *in vitro* lactylation (Lacty). **(K)** Molecular docking simulation predicting the structural interaction interface, highlighting the ANT2 K92 residue within the binding pocket of PGAM5. *** P < 0.05, ** P < 0.01, *** P < 0.001, **** P < 0.0001; ns, not significant.**

**Figure S4. (A, B)** Relative cell viability (A) and quantification of relative LDH release (B) in BT-549 cells under cisplatin stress (0-40 μM, 48 h). Cells expressing wild-type ANT2 (ANT2-WT) or the lactylation-mimetic mutant (ANT2-K92Q) were subjected to shRNA-mediated KAT8 knockdown. Data are presented as mean ± SEM. Statistical significance among the groups across multiple concentrations was determined by two-way ANOVA followed by Tukey's multiple comparisons test. **(C, D)** Quantification of relative LDH release (C) and relative cell viability (D) in BT-549 cells under cisplatin stress (0-40 μM, 48 h). Cells with shRNA-mediated PGAM5 knockdown (shPGAM5) were either treated with exogenous sodium lactate (NALA) or subjected to KAT8 overexpression. Data are presented as mean ± SEM. Statistical significance among the four experimental groups across multiple concentrations was determined by two-way ANOVA followed by Tukey's multiple comparisons test. **(E)** Schematic illustration of the Perceived Stress Scale (PSS-10) scoring system utilized to clinically stratify the breast cancer patient cohort into distinct stress levels. **(F)** Immunoblotting analysis demonstrating the upregulation of LDHA protein expression in tumor cells treated *in vitro* with the stress-related neurotransmitter norepinephrine (NE). β-Actin served as the loading control. **(G)** Immunoblotting analysis with a Pan-Kla antibody revealing that treatment with the competitive peptide P3, or its site-directed mutant P3(R), does not alter the global cellular lactylation landscape. **(H)** Representative immunofluorescence images (left) and quantification of the CD8^+^ positive area (right) in orthotopic 4T1 tumors. Cohorts include control, chronic stress (CS), P3 peptide treatment, and combined CS with P3 treatment. Scale bars,200 μm. Statistical significance among the four groups was determined by one-way ANOVA followed by Tukey's multiple comparisons test. **(I)** Representative flow cytometry contour plots assessing the proportion of infiltrating CD8^+^ T cells in the tumors from the indicated *in vivo* treatment groups in (H). **(J)** Quantitative analysis of CD8^+^ T cell infiltration (% of CD8^+^CD45^+^ cells) corresponding to the flow cytometry data in (I). Data are presented as mean ± SD. Statistical significance among the four groups was determined by one-way ANOVA followed by Tukey's multiple comparisons test. **(K)** Representative hematoxylin and eosin (H&E) stained sections of major organs (lung, heart, liver, kidney, spleen) from control and P3-treated mice, indicating no obvious systemic histological toxicity. **Scale bars, μm. * P < 0.05, ** P < 0.01, *** P < 0.001, **** P < 0.0001; ns, not significant.**
